# *Caenorhabditis elegans* Male Copulation Circuitry Incorporates Sex-Shared Defecation Components To Promote Intromission and Sperm Transfer

**DOI:** 10.1534/g3.116.036756

**Published:** 2016-12-27

**Authors:** Brigitte LeBoeuf, L. Rene Garcia

**Affiliations:** Department of Biology, Texas A&M University, College Station, Texas 77845

**Keywords:** G-CaMP, channelrhodopsin2, wiring diagram, neuropeptides, intestine

## Abstract

Sexual dimorphism can be achieved using a variety of mechanisms, including sex-specific circuits and sex-specific function of shared circuits, though how these work together to produce sexually dimorphic behaviors requires further investigation. Here, we explore how components of the sex-shared defecation circuitry are incorporated into the sex-specific male mating circuitry in *Caenorhabditis elegans* to produce successful copulation. Using behavioral studies, calcium imaging, and genetic manipulation, we show that aspects of the defecation system are coopted by the male copulatory circuitry to facilitate intromission and ejaculation. Similar to hermaphrodites, male defecation is initiated by an intestinal calcium wave, but circuit activity is coordinated differently during mating. In hermaphrodites, the tail neuron DVB promotes expulsion of gut contents through the release of the neurotransmitter GABA onto the anal depressor muscle. However, in the male, both neuron and muscle take on modified functions to promote successful copulation. Males require calcium-dependent activator protein for secretion (CAPS)/*unc-31*, a dense core vesicle exocytosis activator protein, in the DVB to regulate copulatory spicule insertion, while the anal depressor is remodeled to promote release of sperm into the hermaphrodite. This work shows how sex-shared circuitry is modified in multiple ways to contribute to sex-specific mating.

Sexual dimorphism is achieved not only through sex-specific circuitry but also through sexual modification of isomorphic circuitry. However, unlike the more readily identifiable phenotypic sex differences that are required for copulation success, less is known how sex differences impact shared circuitry. Most recent work has focused on delineating the different roles of shared brain regions to promote sex-specific aspects of mating behavior in mammals (Bayless and Shah 2016). For example, sensory organs in the mouse head display an equal number of chemoreceptors, but produce sexually distinct behaviors; this difference is a result of underlying differences in function between sex-shared brain regions, though how this circuit functions still remains to be elucidated (Bayless and Shah 2016).

Similar to mammals, sexually dimorphic behavior generated by sex-shared circuitry has focused on the brains of *Drosophila melanogaster*. One of the better characterized examples is the role of the transcription factor *fruitless* (*fru*) in masculinizing behavior circuits (Ito *et al.* 1996; Ryner *et al.* 1996). *fru* is involved in establishing the sexually dimorphic mAL cluster in the *Drosophila* brain, which contains five neurons in females and 30 neurons in males with sexually dimorphic processes (Kimura *et al.* 2005). Activity of the male-specific *fru*-positive mAL neurons prevents males from engaging in inappropriate male–male courtship (Kallman *et al.* 2015). Additionally, male courtship patterns depend upon a *fru*-dependent male-specific process in the mAL neurons (Ito *et al.* 2016). The study of the mAL cluster indicates how number, morphology, and gene expression contribute to sexually dimorphic mating behavior.

Similar to *Drosophila*, sex-shared head neurons in *Caenorhabditis elegans* can be modified to produce specific sex-related behaviors. One pair of sensory neurons produce sex-specific responses to attractive odorants in a way that allows well-fed males to ignore feeding odorants and to mate (Lee and Portman 2007; Ryan *et al.* 2014). These same sensory neurons also promote male attraction to mating pheromones (White *et al.* 2007). A different pair of sensory neurons is responsible for suppressing attraction in hermaphrodites (White and Jorgensen 2012). These important studies demonstrate the efficiency of exploring the role of sex-based genetic identity of individual cells or groups of cells in model organisms.

In addition to the sexual dimorphism present in the sensory system in the head, there are also differences in the defecation system located primarily in the tail. *C. elegans* defecation is a rhythmic behavior occurring at ∼50 sec intervals when the worm is on food and is divided into three steps: posterior body wall muscle contraction (pBoc), anterior body wall muscle contraction (aBoc), and expulsion (exp) (Croll 1975; Thomas 1990). The intestine acts as the central pacemaker, generating a calcium wave that moves from posterior to anterior and initiates the different defecation cycle steps (Dal Santo *et al.* 1999; Teramoto and Iwasaki 2006). The first step, pBoc, which concentrates the intestinal contents in the anterior of the worm, is initiated by the calcium increase in the posterior intestine that causes H^+^ to be released in the space between the intestine and body wall muscle. The change in pH activates receptors that produce the muscle contractions (Beg *et al.* 2008). The moving calcium wave then generates the aBoc, which transfers the pressurized gut contents from anterior to posterior. This step is dependent upon neuropeptide release, as worms lacking CAPS, a calcium-activated protein that promotes dense core vesicle release, display greatly reduced numbers of aBocs (Speese *et al.* 2007). Finally, the expulsion step is triggered by the release of a neuropeptide, NLP-40, from the intestine onto the DVB motor neuron, activating the Gα_s_-coupled AEX-2 receptor (Wang *et al.* 2013). The DVB then releases GABA onto the enteric muscle, resulting in their contraction and the expulsion of gut contents (McIntire *et al.* 1993b).

While this system is present in hermaphrodites and males, multiple lines of evidence indicate that it is sexually dimorphic. A comparison of constipation phenotypes caused by mutations in genes regulating defecation between the two sexes revealed marked differences (Reiner and Thomas 1995). Structural changes are also apparent in two enteric muscle, the sphincter and anal depressor. The hermaphrodite sphincter has a ventral attachment with the anal depressor muscle and keeps the anus closed, while the male sphincter has a dorsal attachment to the hypodermis and the anus remains open (Reiner and Thomas 1995). The hermaphrodite anal depressor is dorsally attached to the hypodermis and ventrally to the rectum, containing a dorsal–ventral sarcomere, and promotes rectum opening (Thomas 1990; Reiner and Thomas 1995). In the male, this muscle is rearranged to contain an anterior–posterior sarcomere associated with structures required for successful copulatory organ insertion into the hermaphrodite vulva (Sulston *et al.* 1980; Chen and García 2015). Synaptic connectivity changes along with the structural modifications, as the GABAergic DVB neuron no longer synapses the enteric muscle but instead displays extensive connectivity with the male mating circuitry (Figure 1, A and B) (White *et al.* 1986; Jarrell *et al.* 2012).

**Figure 1:**
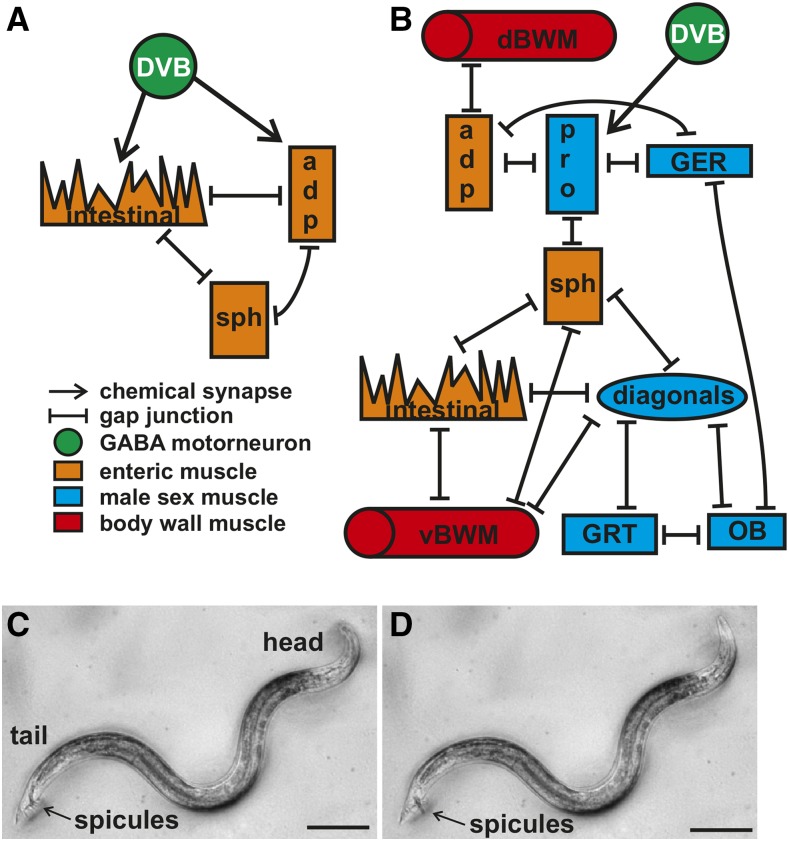
Defecation and male mating circuitry. (A) Connectivity of defecation circuit in the hermaphrodite. Adapted from White *et al.* (1986). (B) Connectivity of the defecation and mating circuitry in the male. Adapted from Jarrell *et al.* (2012). (C and D) Males protract their copulatory spicules following defecation. Dorsal is to the top, anterior to the right. Scale bar = 100 µm. (C) Image of a free-moving male with his spicules inside the cloaca. (D) Image of the male protracting his spicules following expulsion. adp, anal depressor; dBWM, dorsal body wall muscle; GABA, γ-aminobutyric acid; ger, gubernaculum erector; grt, gubernaculum retractor; ob, obliques; pro, protractor; sph, sphincter; vBWM, ventral body wall muscle.

The change in defecation components in the male tail coincides with the development of the structures and circuitry necessary for male copulation (Sulston *et al.* 1980; Chen and García 2015). Both the neuronal and muscle complement are greatly expanded in the male, as 83 new neurons and 40 new muscle are generated, all involved in mating behavior (Jarrell *et al.* 2012; Sammut *et al.* 2015). Additional structures include the copulatory spicules, necessary for the male to first breach and then keep open the hermaphrodite vulva, and the gubernaculum, which contains the copulatory structures. These structures in the male tail help him accomplish the stereotyped steps of mating, including searching for, spicule prodding at, and spicule insertion into the vulva, and finally sperm transfer. To search for the vulva, males place their tails on the hermaphrodite cuticle and initiate backward movement in a process controlled by sex-specific sensory rays and the sex-shared command interneuron AVA (Koo *et al.* 2011; Sherlekar *et al.* 2013). The vulva is sensed by the hook sensilla and the postcloacal sensilla, which are located immediately to the fore and rear of the cloaca opening, respectively (Liu and Sternberg 1995; Barr and Sternberg 1999). These neurons act together with sex muscle to create the feedback loop necessary to control spicule prodding at the vulva (Liu *et al.* 2011; Correa *et al.* 2012). Once the vulva has been breached, full spicule insertion is achieved through acetylcholine secretion from the SPC neurons onto the spicule protractor muscle (Garcia *et al.* 2001). Sperm is then released from the valve, travels down the vas deferens, and exits through the cloaca into the uterus (Schindelman *et al.* 2006).

While previous work has primarily focused on how defecation or mating is integrated with movement (Sherlekar *et al.* 2013; Nagy *et al.* 2015), itself a sexually dimorphic behavior (Mowrey *et al.* 2014), here we focus on how defecation and mating are integrated with each other. Males stop defecating once the mating program is initiated, and we determined how various defecation components are utilized to promote mating. While the intestine and intestinal muscle function similarly during defecation in both sexes, in males the anal depressor activity is delayed. Additional activity is seen in the sex muscle, occasionally leading to spicule protraction immediately following expulsion of gut contents. During mating, these same muscle are active during spicule insertion and sperm movement. We show that the sexually modified anal depressor modulates sex organ positioning, while neuropeptide signaling from the DVB neuron promotes spicule insertion. We highlight how sexual dimorphism can be achieved using multiple mechanisms, including structural and signaling changes.

## Materials and Methods

### Strains

All strains were maintained on NGM agar seeded with *Escherichia coli* OP50, according to methods stated in (Brenner 1974). Since *C. elegans* is a hermaphroditic species that produces males at a low rate, all strains used contain *him-5*(*e1490*) for their high instance of males (Hodgkin *et al.* 1979). Strains used in this study are: *inx-16*(*ox144*) (Peters *et al.* 2007) on LGI, *pha-1*(*e2123*) (Schnabel and Schnabel 1990) and *unc-64*(*e426*) (Brenner 1974) on LGIII, *unc-31*(*e169*) (Avery *et al.* 1993) and *him-8*(*e1489*) (Hodgkin *et al.* 1979) on LGIV, *egl-3*(*ok979*) (*C. elegans* Gene Knockout Consortium) on LGV, and *lite-1*(*ce314*) (Edwards *et al.* 2008), *aex-2*(*sa3*) (Mahoney *et al.* 2008), and *pbo-4*(*ok583*) (Beg *et al.* 2008) on LGX.

Transgenic strains include: rgEx326[P*tph-1*:CFP], rgEx327[P*tph-1*:YFP], rgEx742[P*gtl-1*:G-CaMP3:SL2:dsRed+P*unc-103*E:G-CaMP3:SL2:dsRed], rgEx813[P*aex-2*:G-CaMP6:SL2:dsRed+P*unc-103*J:ChR2:YFP], rgEx760[QUAS:YFP:*unc-31*], rgEx759[P*rab-3*:YFP:*unc-31*], rgEx809[P*aex-2*:YFP:ChR2], and rgEx780[P*unc-103*J:YFP:*unc-31*].

### Plasmid construction

#### Promoters:

Previously published plasmids and their references containing promoter constructs are listed in Table 1. Primers used in this study are listed in Table 2. We amplified 1.2 kb upstream of the *rab-3* start codon from genomic DNA using primers fprab-3 and prab-3r. We used Invitrogen BP clonase to recombine the PCR product with pDG15 (Reiner *et al.* 2006) to generate plasmid pBL363. We amplified 2 kb upstream of the *aex-2* start codon using primers fPaex-2 and Paex-2r. We used Invitrogen BP clonase to recombine the PCR product with pDONR to generate plasmid pBL348. To create P*unc-103*J, we removed part of the Punc-103E promoter from pXG31[P*unc-103*E:YFP:actin] (Guo *et al.* 2012) using primers fpXG31 and pXG31r. P*unc-103*J starts with sequence 5′-GTAAGTGGAACTTTT-3′ and ends with sequence 5′-TCATCGACTGGAGCA-3′. We ligated the PCR product to create pLR343. We recombined pLR343 with pDONR using BP clonase to create plasmid pBL373.

**Table 1:** Entry vectors used in this study

Promoter	Plasmid	Expression	Reference
Pgtl-1	pBL63	Intestine	Teramoto *et al.* (2005) and LeBoeuf *et al.* (2007)
Paex-2	pBL348	Enteric muscle, DVB	Mahoney *et al.* (2008)
Punc-103E	pLR21	Sex muscle	Reiner *et al.* (2006)
Prab-3	pBL363	Panneuronal	Nonet *et al.* (1997)
Punc-103J	pBL373	AVL, DVB	E. Jorgensen, personal communication
QUAS	pCJ138	None	Wei *et al.* (2012) and Jee *et al.* (2016)

**Table 2:** Primers

Primer Name	Primer Sequence
func31cDNA	GATGAACTATACAAAATGTCGAATGTTTCAAAGCCGATAATGCAAAATTC
unc31cDNAmidr	ATTCTACGAATGCTA CATGATCGAAATTAATCGAATCAGCCTGAATC
fpgw322inf	TAGCATTCGTAGAATTCCAACTGAGC
pgw322infr	TTTGTATAGTTCATCCATGCCATGTGTAATC
pBL360r	CATGATCGAAATTAATCGAATCAGCCTGAATC
fmidunc31cDNA	TTAATTTCGATCATGACCATTTTTATTCCGATG
unc31cDNAr	ATTCTACGAATGCTAATGTTTTCGTATACCTTCTTGAATATGAGAATTTGACTC
fprab-3	GGGGACAAGTTTGTACAAAAAAGCAGGCTGATCTTCAGATGGGAGCAGTG
prab-3r	GGGGACCACTTTGTACAAGAAAGCTGGGTCTGAAAATAGGGCTACTGTAGATTTATTTTA
fPaex-2	GGGGACAAGTTTGTACAAAAAAGCAGGCTCTAGAACACTTTGGGTAACGTGGTA
Paex-2r	GGGGACCACTTTGTACAAGAAAGCTGGGTTCTGAAATTTGTTTTGTTAGAAAAAAGGTCG
fpXG31	TGCTCCAGTCGATGATGACTTTGGGATCGCAGATGATGATGG
pXG31r	TACCCAGCTTTCTTGTACAAAGTGGTTCGATCTAGAGGA

#### unc-31 cDNA:

For YFP:*unc-31* cDNA plasmid construction, we obtained a strain containing YFP:unc-31 on the ceEx117 transgene from Ken Miller (Charlie *et al.* 2006). After worm lysis to obtain DNA, we PCR amplified the first half of the *unc-31* cDNA using primers func31cDNA and unc31cDNAmidr. This PCR product was combined with pGW322YFP linearized using primers fpgw322inf and pgw322infr using the Clontech Infusion kit to generate plasmid pBL360. pBL360 was linearized using primers pBL360r and fpgw322inf and combined with the second half of unc-31 cDNA that was PCR amplified using primers fmedunc31cDNA and unc31cDNAr to generate plasmid pBL367. The plasmid was sequenced to ensure no mutations. pBL367 contains an Invitrogen Gateway Reading Frame Cassette, allowing for easy transfer of promoters in front of the YFP:unc-31 sequence. pBL367 and all other Gateway Destination Vectors used in this study are listed in Table 3. Using LR clonase, we transferred QUAS, P*rab-3*, and P*unc-103*J to pBL367 to create plasmids pBL370, pBL371, and pBL374, respectively.

**Table 3:** Destination vectors used in this study

Gateway Destination Vector	Contains	Reference
pLR289	RfC:G-CaMP3:SL2:dsRed	Correa *et al.* (2012)
pLR305	RfC:G-CaMP6M:SL2:dsRed	LeBoeuf and Garcia (2012)
pBL367	RfC:YFP:unc-31	This paper
pBL248	RfC:ChR2:YFP	This paper

#### G-CaMP:

pLR289(P*unc-103*E:G-CaMP3:SL2:dsRed) construction was described in LeBoeuf *et al.* (2014). pBL260(P*gtl-1*:G-CaMP3:SL2:dsRed) was creating by using an LR reaction to recombine pBL63(Pgtl-1) (LeBoeuf *et al.* 2007) with pBL279(G-CaMP3:SL2:dsRed) (Correa *et al.* 2012). pBL352(P*aex-2*:G-CaMP6M:SL2:dsRed) was created by LR reaction to recombine pBL348(P*aex-2*) with pLR305(G-CaMP6M:SL2:dsRed).

#### Channelrhodopsin2 (ChR2):

The ChR2 plasmid pLR167 described in Liu *et al.* (2011) had YFP removed, and instead we added YFP with more introns from pGW322YFP in an effort to make the protein expression more abundant. This plasmid is pBL248 and contains a Gateway Reading Frame Cassette. pBL248 was recombined with pBL348 (P*aex-2*) using LR Clonase to generate pBL391. pBL248 was also recombined with pBL373 (P*unc-103*J) to make pBL381.

### Transgenic worm generation

All injection mixtures contained 50 ng/µl of pBX1[*pha-1*(wild-type)] (Schnabel and Schnabel 1990). This allowed us to inject into *pha-1*(*ts*) hermaphrodites and incubate injected animals at the nonpermissive temperature. Only progeny carrying an extrachromosomal array survived to adulthood. Injection mixtures additionally contained pUC18 as carrier DNA. Injected amounts are listed in Table 4.

**Table 4:** Plasmids used to make extrachromosomal arrays

Plasmid Name	Plasmid Contains	Injection Amounts (ng/µl)
pBL260	Pgtl-1:G-CaMP3:SL2:dsRed	50
pBL352	Paex-2:G-CaMP6M:SL2:dsRed	50
pLR289	Punc-103E:G-CaMP3:SL2:dsRed	100
pBL370	QUAS:YFP:unc-31	30
pBL371	Prab-3:YFP:unc-31	50
pBL374	Punc-103J:YFP:unc-31	50
pBL391	Paex-2:ChR2:YFP	70
pBL381	Punc-103J:ChR2:YFP	70

### Behavior assays

#### Defecation:

Worms were isolated by gender at L4 the day before and allowed to mature overnight. One worm was then placed on a fresh OP50 *E. coli* lawn and recorded using an Olympus BX51 microscope and Hamamatsu ImagEM Electron multiplier (EM) CCD camera.

#### Mating:

10 µl of saturated *E. coli* OP50 was spotted to an NGM plate and allowed to dry. Fifteen 2-d-old *unc-64*(*lf*) hermaphrodites were transferred to the OP50 and allowed to incubate for at least 1 hr. The OP50 + NGM + hermaphrodites were then placed on a microscope slide. The behavior of one male at a time was recorded using an Olympus BX51 microscope and Hamamatsu ImagEM Electron multiplier (EM) CCD camera. Males were given 10 min to insert their spicules into the hermaphrodite uterus, after which the mating trial was terminated. Spicule insertion % was determined by calculating the number of males that were successfully able to insert their spicules within 10 min *vs.* the total number of males tested. Sperm release from the valve % was determined using the total number of males that inserted. All males that were able to release some sperm from the valve were counted as successful whether or not they were able to release sperm from the cloaca. Sperm release measures all males that successfully transferred sperm into the uterus *vs.* the total number of males that inserted their spicules. The total amount of time that males’ spicules were inserted measures from when they inserted their spicules until they either retracted their spicules into their cloaca or removed the still-protracted spicules from the uterus. The competition assay was done as described in LeBoeuf *et al.* (2011).

### Behavior assay analysis

Statistical analysis was done using GraphPad Prism (GraphPad Software, Inc., La Jolla, CA). We have previously determined that, since mating behavior can vary from day to day, it is necessary to perform same-day controls for each experiment (Guo *et al.* 2012). For the assay of mating behavior for the defecation mutants, each mutant was compared to a control strain done at the same time. The wild-type data collected at the same time as the *unc-31*(*lf*) mutant males was then normalized to 100. The other mutants were then normalized accordingly. Nonnormalized data are as follows for insertion: control 97% (*n* = 29), *unc-31*(*lf*) 67% (*n* = 29); control 79% (*n* = 14), *aex-2*(*lf*) 100% (*n* = 15); control 81% (*n* = 21), *inx-16*(*lf*) 50% (*n* = 18); control 50% (*n* = 12), *pbo-4*(*lf*) 67% (*n* = 12); control 100% (*n* = 21), *egl-3*(*lf*) 73% (*n* = 22). Both *unc-31*(*lf*) and *egl-3*(*lf*) display statistically significant decreases in spicule insertion using Fisher’s exact test, while *inx-16*(*lf*) does not.

### Still imaging and analysis

Still images of the intestinal lumen were taken with an Olympus IX81 microscope, csu-xi Yokogawa spinning disk, and Andor iXon EM CCD camera. They were analyzed using Metamorph software (version 7.8.0.0, Molecular Devices, Sunnyvale, CA). Images of a micrometer taken at the same magnification were used to calibrate the measuring tool. This tool was then used to determine the width of the intestinal lumen.

### G-CaMP imaging and analysis

Calcium imaging and analysis was done as described in LeBoeuf *et al.* (2014). For defecation analysis, males and hermaphrodites were put individually on lawns of *E. coli* OP50 and allowed to move freely while they were being recorded. For males that needed to be restricted, we used 8% noble agar pads plus Polybead polystyrene 0.1 µm microspheres (Polysciences, Warrington, PA) (Kim *et al.* 2013) or 3% noble agar plus S-basal pads containing 250 µg/ml abamectin (Sigma-Aldrich, St. Louis, MO). Images of a micrometer taken at the same magnification were used to calibrate the measuring tool. This tool was then used to determine the width of the posterior male tail from the dorsal cuticle to the cloaca.

### Cell ablations

Cell ablations were performed as described in Bargmann and Avery (1995). We used a Micropoint 337-NDS-USAS laser (Photonic Instruments, St. Charles, IL) attached to an Olympus BX51 microscope (Olympus, PA). Males were placed on 2.5% noble agar pads dissolved in M9 plus 12 mM NaN_3_. Half of the males had their anal depressors ablated while the others were used as nonoperated controls.

### Data availability

Plasmids and strains are available upon request.

## Results

### C. elegans male mating and defecation circuitry are functionally connected

*C. elegans* males’ copulatory spicules remain inside the cloaca except when they are inserted into the hermaphrodite vulva to facilitate sperm transfer. Previous work noted that free-moving males occasionally protract their copulatory spicules in the absence of mating cues. This brief protraction can, on rare occasions, become permanent, incapacitating the males’ ability to mate (Garcia and Sternberg 2003; Jobson *et al.* 2015). We asked if this nonmating associated spicule protraction is associated with defecation. *C. elegans* defecation occurs in three steps: (1) anterior body wall muscle contraction (aBoc), (2) posterior body wall muscle contraction (pBoc), and (3) expulsion (exp) (Thomas 1990). We reasoned that spicule protraction is most likely to occur coinciding with the exp step, as the enteric muscle that regulate expulsion are connected to the male sex muscle via gap junctions (Jarrell *et al.* 2012). We found this to be the case, as males protract their spicules 41 ± 17% of the time following an exp (*n* = 37 total exps measured from six individual males; each male performed at least five exps; every male displayed at least one protraction and no male had higher than a 57% protraction rate following an exp) (Figure 1, C and D). This result suggests that a functional connection exists between the male mating and defecation circuitry.

**Figure 2:**
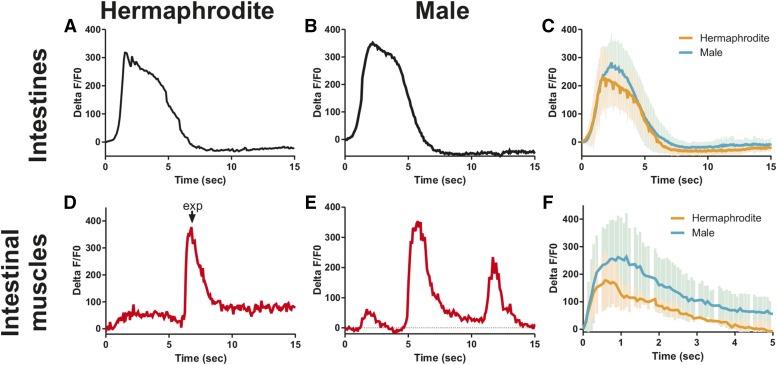
Ca^2+^ transients increase in both sexes in the intestine and intestinal muscle during defecation. (A–C) %ΔF/F0 traces for the intestine in one representative hermaphrodite (A), one representative male (B), and the average of five worms for both sexes (C). (D–F) %ΔF/F0 traces for the intestinal muscle in one representative hermaphrodite (D), one representative male (E), and the average of five worms for both sexes (F). Error bars in (C) and (F) represent SD. exp, expulsion; this indicates when the hermaphrodite expelled gut contents from her rectum.

To address this issue further, we expressed the calcium indicator G-CaMP in the different defecation system components, including the intestine, intestinal muscle, and anal depressor. The intestine are the pacemaker for the defecation motor program: the increase of Ca^2+^ transients in the posterior intestine initiates the defecation cycle (Teramoto and Iwasaki 2006). Several studies have used intestinal G-CaMP to highlight the importance of the posterior-to-anterior Ca^2+^ wave in coordinating defecation (Espelt *et al.* 2005; Teramoto and Iwasaki 2006; Peters *et al.* 2007; Wagner *et al.* 2011). Here, we focus on the posterior intestine that can be imaged during male mating. We allowed worms to move freely on bacteria and simultaneously recorded the fluorescent levels in G-CaMP (green) and an unchanging control, red fluorescent protein from *Discosoma* (red). We expressed the fluorescent calcium sensor in the intestine using the *gtl-1* promoter (Teramoto *et al.* 2005). Similar to previously published results in the hermaphrodite (Espelt *et al.* 2005; Teramoto and Iwasaki 2006), we find that the posterior intestine displays an increase in Ca^2+^ for ∼5 sec, initiating the defecation cycle (Figure 2A). The male intestine shows an identical Ca^2+^ increase to initiate the defecation cycle (Figure 2, B and C). Thus, the role of the intestine in initiating the rhythmic behavior is unmodified in the male.

**Figure 3:**
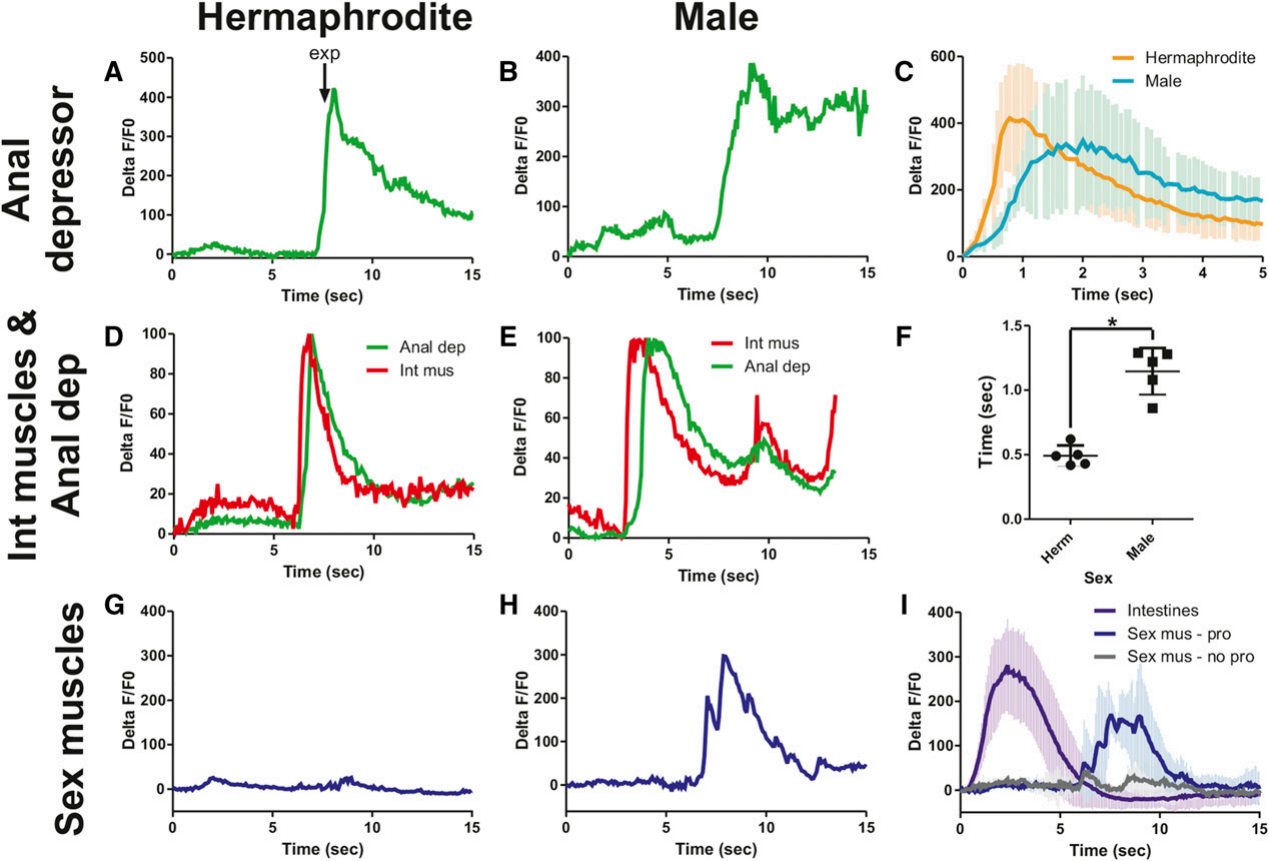
Ca^2+^ transient activity is altered in the male anal depressor. (A–C) %ΔF/F0 traces for the anal depressor in one representative hermaphrodite (A), one representative male (B), and the average of five worms for both sexes (C). (D and E) Normalized Ca^2+^ transient traces for the anal depressor and intestinal muscle in one representative hermaphrodite (D) and one male (E). The lowest %ΔF/F0 was set at 0%, the highest at 100%. (F) The time between the %ΔF/F0max for the intestinal muscle and anal depressor. Each dot represents one individual. Bar is the mean. * *P* < 0.05, Fisher’s exact test. (G) %ΔF/F0 traces for the vulva muscle in one representative hermaphrodite. (H) %ΔF/F0 traces for the sex muscle in one representative male. (I) Average %ΔF/F0 trace for the intestine (purple, *n* = 5) and sex muscle (blue, *n* = 4 and gray, *n* = 2). Sex mus – pro = sex muscle protraction: the %ΔF/F0 traces for males that protract their spicules. Sex mus – no pro = sex muscle – no protraction: the %ΔF/F0 traces for males that protract their spicules. Error bars in (C), (F), and (I) represent SD. Anal dep, anal depressor; exp, expulsion; Herm, hermaphrodite; Int mus, intestinal muscle.

In hermaphrodites, the increase in Ca^2+^ intestinal signaling results in the release of the neuropeptide NLP-40, which activates the DVB motor neuron in the tail (Wang *et al.* 2013). The DVB then releases GABA onto the intestinal muscle and anal depressor, causing them to contract and expel gut contents: the exp step of the defecation cycle (McIntire *et al.* 1993b). However, corresponding Ca^2+^ changes have not been reported in these muscle for either sex. We used the *aex-2* promoter to express our fluorescent calcium indicator in these muscle (Mahoney *et al.* 2008). As expected, we find an increase in fluorescent levels corresponding to exp in the intestinal muscle of both sexes (Figure 2, D–F). Like the calcium transient dynamics in the intestine (Figure 2C), the calcium changes in the intestinal muscle in the hermaphrodite match the male closely (Figure 2F). In contrast, while the calcium transient changes start at the same time in both sexes in the anal depressor, they increase faster in the hermaphrodite (Figure 3, A–C). Thus, there is a larger separation between the intestinal muscle and anal depressor calcium peaks in the male than the hermaphrodite (Figure 3, D and E). The calcium transient peak in the male occurs 0.65 sec after the hermaphrodite (Figure 3F, *P* = 0.007, Fisher’s exact test). Unlike the hermaphrodite, the adult male anal depressor does not synapse the other enteric muscle, the intestinal muscle, or sphincter, is not synapsed by the DVB, and is not necessary for defecation (Figure 1, A and B) (White *et al.* 1986; Reiner and Thomas 1995; Emmons 2005). Given this, it is surprising that the male anal depressor displays any calcium transient increase during defecation. However, the anal depressor is still indirectly linked to the defecation circuitry through gap junctions with the male protractor muscle that are also synapsed by the DVB (Figure 1B). Therefore, this muscle could be receiving residual signaling from these sources, causing Ca^2+^ increase during defecation. Nevertheless, the different calcium transient dynamics between the sexes indicate that this muscle is playing a different role.

In males in both the intestinal muscle and anal depressor, a second smaller increase in calcium transients is observed (Figure 2E and Figure 3E). Given that male defecation induces spicule protraction and that many reciprocal connections exist between male mating and defecation circuitry (Figure 1B) (Jarrell *et al.* 2012), we reasoned that this second Ca^2+^ increase could be due to activity in the sex muscle. To test this hypothesis, we expressed G-CaMP in the sex muscle using the *unc-103*E promoter (Reiner *et al.* 2006; Gruninger *et al.* 2008). As expected, we found that hermaphrodite sex muscle displayed no activity during defecation (Figure 3G). Male sex muscle show an increase in calcium levels following an exp that corresponds with spicule protraction (Figure 3, H and I). We did not observe calcium increases when no spicule protraction occurred (Figure 3I). Thus, while hermaphrodite and male defecation show similar kinetics, males have additional components that represent the incorporation of the defecation system into the mating circuitry.

**Figure 4:**
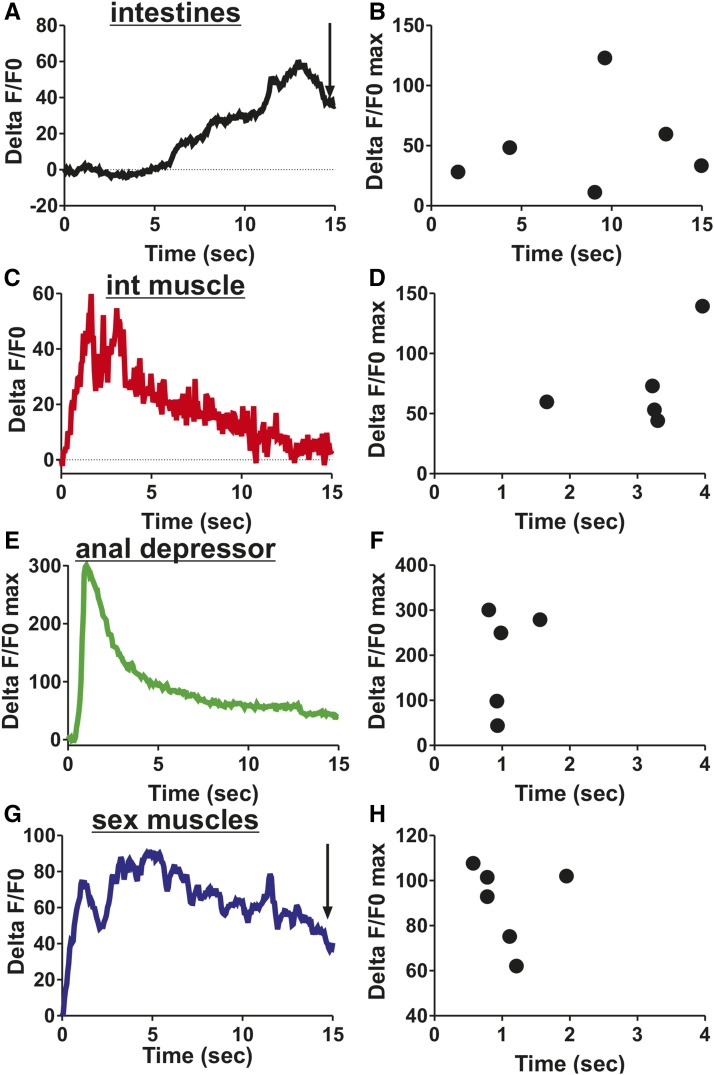
Defecation circuit components are active during male mating. Ca^2+^ traces begin when the male inserts his spicules into the hermaphrodite vulva. (A) %ΔF/F0 traces for the intestine in one representative male. (B) %ΔF/F0max following insertion for male intestine. (C) %ΔF/F0 traces for the intestinal muscle in one representative male. (D) %ΔF/F0max following insertion for male intestinal (int) muscle. (E) %ΔF/F0 traces for the anal depressor in one representative male. (F) %ΔF/F0max following insertion for male anal depressor. (G) %ΔF/F0 traces for the sex muscle in one representative male. (H) %ΔF/F0max following insertion for male sex muscle. Ca^2+^ activity in the sex muscle during mating first reported in LeBoeuf *et al.* (2014). (A and G) Arrow represents visible sperm release into the hermaphrodite. (B, D, F, and H) Each dot represents one individual.

Next, we asked which defecation circuitry components were active during male mating. We mated free moving males to paralyzed hermaphrodites and recorded the fluorescent changes in the intestine, intestinal muscle, anal depressor, and sex muscle during spicule insertion and sperm release. The Ca^2+^ transients that occur in the defecation circuit during spicule insertion and sperm release vary considerably from defecation. While intestinal Ca^2+^ initiates the defecation cycle, intestinal Ca^2+^ does not initiate mating. Ca^2+^ transients in the intestine do not change upon insertion, but increase around the time that sperm moves through the vas deferens (Figure 4, A and B). Insertion causes Ca^2+^ transients to increase in the intestinal muscle and anal depressor and then decline though maintaining a higher level than at insertion (Figure 4, C–F). Thus, in contrast to defecation, intestinal Ca^2+^ is not necessary to activate these muscle. Also in contrast to defecation, the anal depressor %ΔF/F0max occurs 2.0 ± 1.1 sec prior to the intestinal muscle (Figure 4, D and F). Additionally, the %ΔF/F0max of the anal depressor occurs 1.0 ± 0.3 sec after insertion (Figure 4F), similar to that of the sex muscle, 1.1 ± 0.5 sec (Figure 4, G and H). However, the Ca^2+^ transient fluctuations seen in other sex muscle do not occur in the anal depressor (Figure 4E) (LeBoeuf *et al.* 2014). Taken together, these results indicate that many components of the intestinal circuitry have activities that correspond with mating behavior and are different from those seen during defecation. The anal depressor, which was structurally changed in the male from an enteric muscle with a dorsal–ventral sarcomere to a mating muscle with an anterior–posterior sarcomere (Chen and García 2015), displays calcium increases during mating that more closely associate with the male sex muscle, while some separation exists between the anal depressor and the intestinal muscle. These functional data are supported by connectivity data, where the anal depressor is no longer electrically or chemically coupled directly to the defecation circuit but instead synapses with mating circuitry components (Figure 1, A and B) (White *et al.* 1986; Jarrell *et al.* 2012).

**Figure 5:**
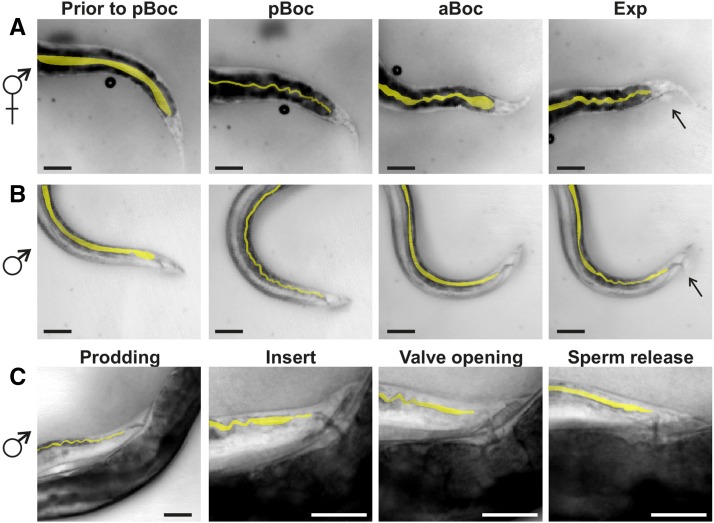
Intestine morphology changes during defecation and mating. Intestinal lumen shape is highlighted in yellow. Dorsal is to the top, posterior to the right. Scale bar = 50 µm. Images of hermaphrodite (A) and male (B) tails during defecation motor program. Arrows in Exp images point to waste being expelled. (C) Images of male tail during male mating steps. aBoc, anterior body wall contractions; exp, expulsion; pBoc, posterior body wall contractions.

The intestinal muscle activity during defecation generates morphological changes in the intestine (Wagner *et al.* 2011). It is possible that the changing muscle activity during mating is having a similar effect on intestinal morphology. Under normal conditions, the hermaphrodite intestine are in a straight line. The pBoc triggers concurrent dorsal and ventral body wall muscle contraction, causing the intestinal lumen to narrow and fold up, forming an accordion shape. These muscle then relax during the aBoc, leading to a partially relaxed intestine. Finally, the enteric muscle contracts, resulting in a scrunched intestine and waste expulsion (Figure 5A). We determined that similar changes occur in the male during defecation, though the most posterior part of the intestine does not display the same maximum contraction phenotype as the hermaphrodite (Figure 5B). We then asked if morphological changes occur in the intestine during mating, despite the fact that the defecation motor program is not utilized. We found that males display slightly scrunched intestine during prodding, a phenotype that leads to a straight, open lumen upon spicule insertion (Figure 5C). During the initiation of sperm movement, some scrunching of the intestine immediately anterior to the posterior region is observed (Figure 5C). Finally, the intestine displays a straight and slightly expanded lumen upon sperm release (Figure 5C). Thus, the male intestine display morphological changes that correspond to an increase in Ca^2+^ transients and sperm movement, indicating that these tissues play a role in the behavior.

### unc-31/CAPS is required for defecation and male mating

Since hermaphrodite and male defecation have similar components, and these two behaviors are linked, we asked if the same mechanisms used in defecation are also applicable to male mating. To address this question, we examined the mating ability of mutants that have disrupted defecation. The defecation cycle is initiated by a calcium wave that is dependent on *inx-16*, a gap junction subunit, for its posterior-to-anterior movement (Peters *et al.* 2007). This Ca^2+^ wave first causes the release of protons from the posterior intestine, a process dependent on *pbo-4*, a putative Na^+^/H^+^ ion exchanger; the release of H^+^ ions causes the dorsal and ventral posterior body wall muscle to contract, resulting in the first step of defecation, the pBoc (Beg *et al.* 2008). The second step of defecation, the aBoc, wherein the dorsal and ventral anterior body wall muscle contract, is at least partially dependent on neuropeptide signaling. Hermaphrodites with a mutation in *unc-31*, a CAPS protein that regulates dense core vesicle release, have a reduced number of aBocs (Speese *et al.* 2007). The final step of defecation, exp, is dependent on GABAergic signaling from the DVB neuron (McIntire *et al.* 1993b). The DVB neuron is activated by the neuropeptide NLP-40 released from the intestine, which then binds and activates the *aex-2* G-protein-coupled receptor located on the DVB (Mahoney *et al.* 2008). The activated DVB then releases GABA onto the enteric muscle, causing the contraction that results in the expulsion of the gut contents (McIntire *et al.* 1993b).

**Figure 6:**
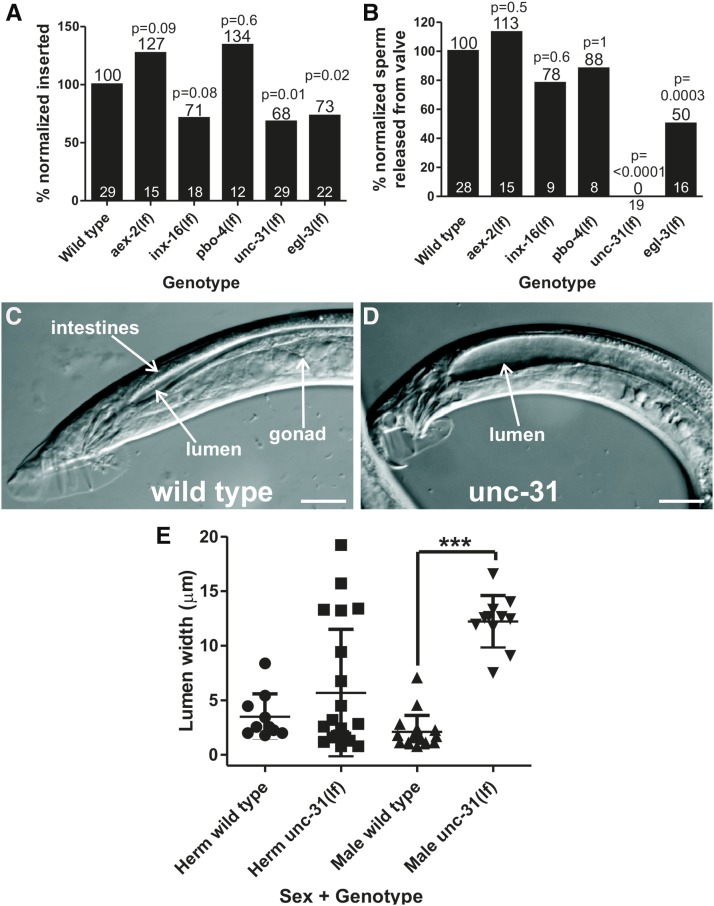
Males lacking *unc-31*/CAPS fail to insert spicules and transfer sperm. (A) Ability of defecation mutant males to insert their spicules into the hermaphrodite vulva. The numerical percentage of wild-type males that inserted their spicules was normalized to 100%. The normalization factor was then applied to the other genotypes. *x*-axis indicates the genotype of the male tested. (B) Ability of males to initiate sperm movement from the seminal vesicle, allowing sperm to move down the vas deferens, out the cloaca, and into the uterus. The numerical percentage of wild-type males that released sperm from the valve was normalized to 100%. The normalization factor was then applied to the other genotypes. *x*-axis indicates the genotype of the male tested. (A and B) The number of males in each assay is at the *x*-axis. The number above each bar is the percentage of males completing a task successfully. The *P* value determined by Fisher’s exact test is given above each bar. *aex-2*(*lf*) and *pbo-4*(*lf*) are not significantly better than wild-type. (C and D) Confocal images of wild-type (C) and *unc-31*(*lf*) (D) adult male tails. Dorsal is to the top, anterior to the right. Scale bar = 20 µM. (E) Measurements of intestine lumen width. *x*-axis indicates the sex and genotype of the worms measured. Each point represents one worm. Line is mean and error bars are SD. *** *P* < 0.0001, Mann–Whitney *U* test. CAPS, calcium-dependent activator protein for secretion; Herm, hermaphrodite.

Since previous work noted severe constipation in male GABA mutants (Reiner and Thomas 1995), we first asked if GABA signaling regulates mating. We examined *unc-25* (glutamic acid decarboxylase) and *unc-47* (transmembrane GABA transporter) mutant males that lack GABA, and found that the lack of coordination caused by GABA deficiency makes it difficult for males to maintain contact with the hermaphrodite. Therefore, we are unable to determine if GABA mutant males’ inability to copulate is due to mating and/or movement defects. When we examined *aex-2* mutant animals, which are specifically disrupted in defecation, we did not see any negative impact on overall mating behavior (Figure 6, A and B). Similarly, mutations disrupting *inx-16* and *pbo-4* did not have a significant impact on mating (Figure 4, A and B). Interestingly, mutation in *unc-31*/CAPS reduced the male’s insertion ability as well as virtually eliminated sperm initiation (Figure 6, A and B); however, the mutant males can perform all of the previous steps of mating successfully. *unc-31*(*lf*) males do not display the process termed sperm initiation; sperm is rarely if ever released from the valve (Figure 6B), the structure that holds the sperm in the seminal vesicle until receiving signals to open and release sperm into the vas deferens (Schindelman *et al.* 2006). We also noticed that the posterior intestinal lumen seemed expanded in males but not hermaphrodites (Figure 6, C and D). To quantify this, we measured the lumen width in worms mounted on agar pads and found that, while mutant hermaphrodites were broadly normal, mutant males displayed a greatly expanded intestine lumen (Figure 6E). The expanded lumen is a constipation phenotype in *C. elegans*, indicating that the expulsion step of the defecation cycle is abnormal (Reiner and Thomas 1995). Finally, we looked at the males’ ability to briefly protract their spicules following defecation, which occurs 41% of the time in wild-type males. In contrast, *unc-31*(*lf*) males only protract their spicules following 10% of defecation cycles (*P* = 0.0059, Fisher’s exact test, 30 total defecation cycles tested from five individual males). Thus, *unc-31* plays an additional role in male defecation compared to the hermaphrodite, while also contributing to multiple steps of male mating.

*unc-31*/CAPS is involved in dense core vesicle release, suggesting that the reason *unc-31* mutants display defects in defecation and mating is due to disrupted neuropeptide release (Speese *et al.* 2007). To confirm this, we tested the mating ability of *egl-3*, which encodes one of four *C. elegans* proprotein convertases, involved in processing neuropeptide precursors (Kass *et al.* 2001). Similar to *unc-31*, *egl-3* was previously reported to have defects in turning behavior; males have difficulty turning from one side of the hermaphrodite to the other as they search for her vulva (Liu *et al.* 2007). We found that *egl-3* males have insertion and sperm initiation defects (Figure 6, A and B). However, these defects are not as severe as *unc-31* defects, suggesting a role for additional proprotein convertases. In summary, molecular pathways involved in pBoc and exp in hermaphrodites are not necessary for male mating, but the neuropeptide signaling coordinated by *unc-31*/CAPS, necessary for the aBoc in hermaphrodite defecation, is important in regulating spicule insertion and sperm release during male mating.

### unc-31/CAPS functions in the defecation-regulating DVB motor neuron

**Figure 7:**
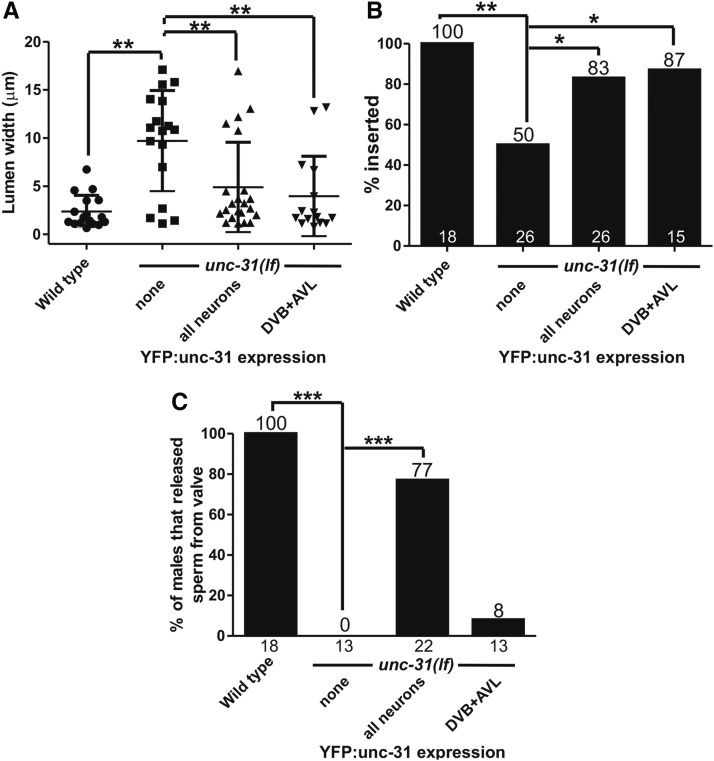
*unc-31* functions in the DVB motor neuron for spicule insertion. *x*-axis indicates genotype and tissues where *unc-31* is rescued. (A) Lumen width of *unc-31*(*lf*) and rescued males. ** *P* < 0.005, Mann–Whitney *U* test. (B) Percentage of males that inserted their spicules into hermaphrodites. Number above bars is the percentage, number at bottom of bars is the *n*. * *P* < 0.05, ** *P* < 0.005, Fisher’s exact test. (C) Percentage of males that released sperm from the valve region into the vas deferens. Numbers above the bars indicate percentage, numbers bellow them indicate *n*. *** *P* < 0.0001, Fisher’s exact test. YFP, yellow fluorescent protein.

Where is *unc-31*/CAPS functioning in the male to promote mating? In the hermaphrodite, *unc-31* is expressed in all neurons plus vulval muscle and the spermatheca (Speese *et al.* 2007); the movement defects in the hermaphrodite were rescued using a pan-neuronal promoter driving a YFP:unc-31 cDNA fusion construct (Charlie *et al.* 2006). We expressed a similar construct in *unc-31*(*lf*) males and analyzed spicule insertion, sperm initiation from the valve, and constipation, to see if these different behaviors are controlled from the same or different tissues. We found that this construct rescues defecation, spicule insertion, and sperm initiation defects (Figure 7, A–C). Thus, neuronal *unc-31* is regulating defecation and male mating in addition to movement.

Does *unc-31* promote defecation, spicule insertion, and sperm initiation from the same or different set of neurons? To address these issues, we expressed *unc-31* cDNA in the DVB neuron from the *unc-103J* promoter (Supplemental Material, Figure S1). GABA, and possibly neuropeptides from the DVB, is released onto the enteric muscle, causing expulsion of the gut contents (Thomas 1990; McIntire *et al.* 1993a,b). Therefore, we initially hypothesized that this construct might restore normal defecation but not male mating. However, we found that while the construct was able to reduce constipation as expected (Figure 7A), it was also able to restore normal spicule insertion (Figure 7B), but had little effect on sperm initiation (Figure 7C). Thus, the DVB retains its sex-shared function in defecation while acquiring a new, *unc-31*-dependent function promoting spicule insertion during male mating. Where UNC-31 functions in the nervous system to promote ejaculation requires further investigation.

### DVB activates the anal depressor in males

Given that CAPS/*unc-31* functions in the DVB to promote spicule insertion, this raises the question of what is the DVB activating. Directly downstream of the DVB in the male are the protractor muscle (Figure 1B) (Jarrell *et al.* 2012). The protractor muscle are responsible for full spicule insertion into the hermaphrodite uterus (Garcia *et al.* 2001). While this process requires the cholinergic motor neuron SPC (Garcia and Sternberg 2003), our data indicate that neuropeptide signaling in the DVB also plays a role in regulating this behavior. Additionally, while no longer directly connected to the DVB, the anal depressor does make electrical connections with the protractors, suggesting that this muscle could still be activated by the DVB. Due to the availability of cell-specific promoters allowing us to visualize the anal depressor without overlaying muscle, we chose to focus on this muscle instead of the protractor muscle, which we cannot visualize alone.

**Figure 8:**
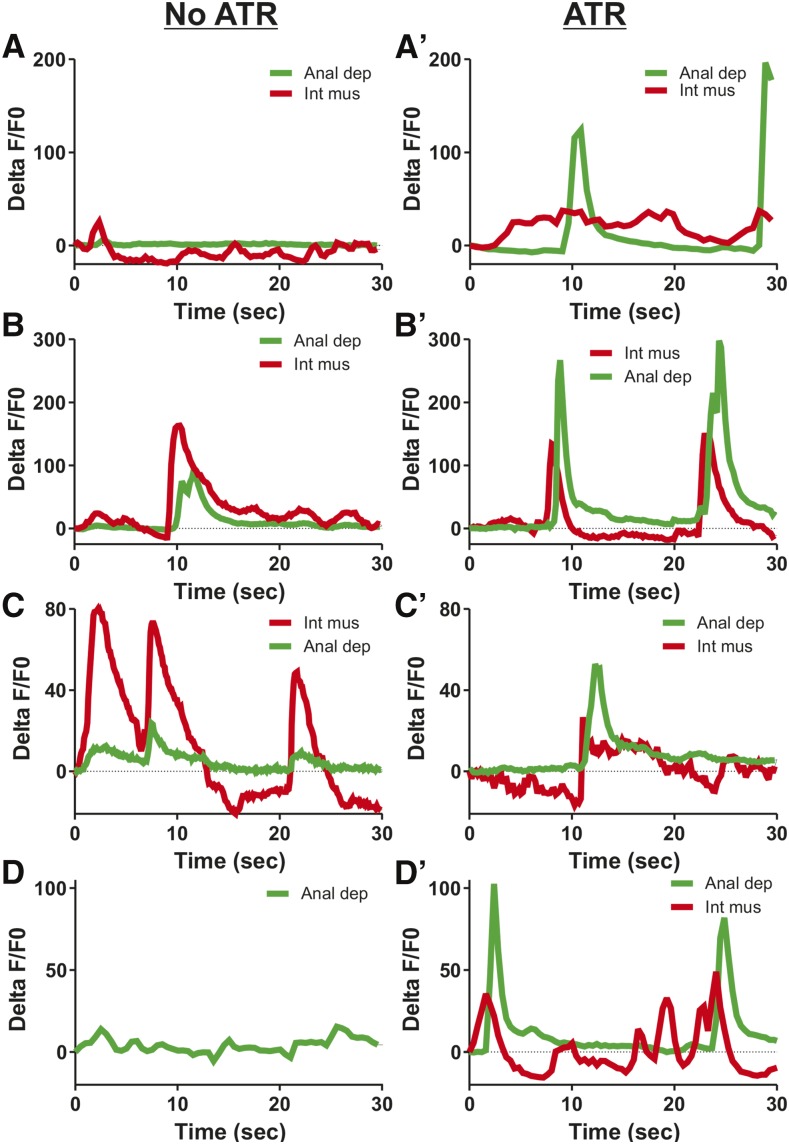
DVB motor neuron can activate the male anal depressor. Anal depressor (Anal dep) and intestinal muscle (Int mus) %ΔF/F0 traces for four individual males. Each male prior to ATR exposure is depicted in A, B, C and D. After ATR incubation, each male's cognate response is depicted in A', B', C' and D'. All recordings taken in the presence of blue light stimulation. ATR, all-*trans* retinol.

We expressed the blue light-activated ChR2 in the DVB and G-CaMP in the anal depressor (Nagel *et al.* 2003, 2005; Mahoney *et al.* 2008). Previously, we showed that using blue light to activate ChR2 expressed in the sex muscle and neurons of free-moving males results in tonic spicule protraction. This protraction is reversible once the blue light stimulation is removed (Liu *et al.* 2011). To test if stimulating the DVB via blue light-activated ChR2 had an effect on free-moving males, we isolated ChR2-expressing L4 males and allowed them to develop overnight to adults before exposing them to all-*trans* retinol (ATR) for at least 1 hr. We then observed free-moving males upon blue light stimulation and noticed transient spicule protraction. Thus, DVB stimulation is able to promote spicule movement. However, stimulating the DVB in this manner did not result in significant disruption of the male movement, making quantification difficult. To address this issue, we immobilized males between a coverslip and high percentage agar and recorded fluorescent changes in the anal depressor. The males were then removed from the agar pads and incubated with ATR for at least an hour, after which the recording session was repeated. While occasional increases in Ca^2+^ occurred in the intestinal muscle prior to ATR exposure, Ca^2+^ transient increases in the anal depressor were observed with less frequency and magnitude (Figure 8). In contrast, following ATR exposure, Ca^2+^ transient increases in the anal depressor were far more common and exceeded the activity in the intestinal muscle (Figure 8). Thus, the DVB is still able to stimulate anal depressor activity despite the loss of direct connectivity.

### The anal depressor fine-tunes spicule position during male mating

While the ChR2 results indicate that the DVB stimulates the anal depressor in the male, it still leaves the question of what role this muscle has during mating. The anal depressor undergoes a structural change in the male from an enteric muscle with a dorsal–ventral sarcomere to a mating muscle with an anterior–posterior sarcomere (Chen and García 2015). This structural change appears necessary for anal depressor function in the male, as previous work has established that males with a feminized anal depressor display many mating defects, including vulva location and insertion behavior (Chen and García 2015). Thus, the rearrangement that occurs between the male larval and adult stages to the anal depressor are necessary for mating success. Additionally, ablating the anal depressor reduces mutant-induced abnormal spicule protraction, suggesting that the muscle is involved in male circuit signaling (Garcia and Sternberg 2003).

**Figure 9:**
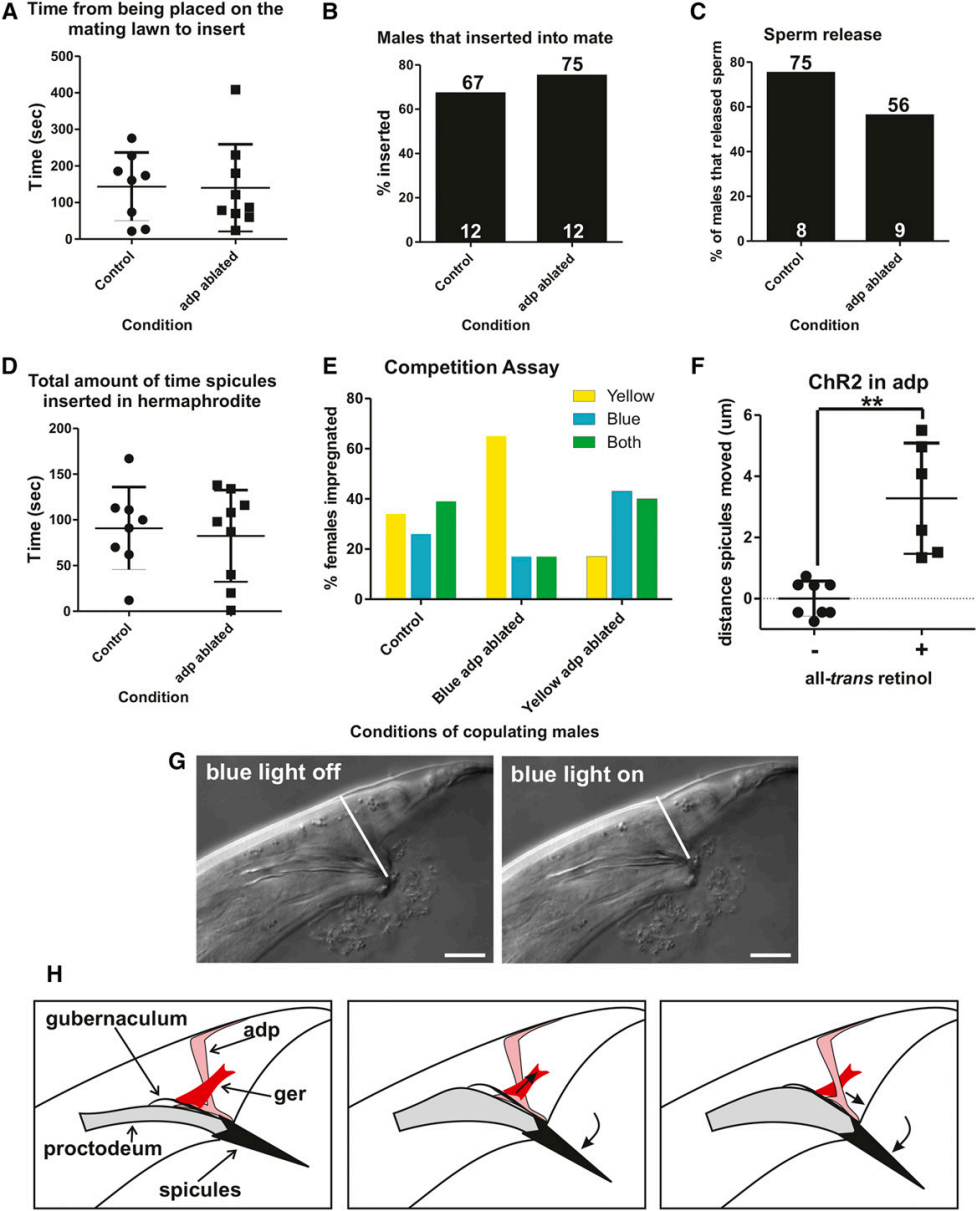
The male anal depressor fine-tunes copulatory organ positioning to promote mating fitness. Numbers at the top of bars indicate percentage, while numbers inside at the bottom of bars indicate the *n*. (A–E) Males with the anal depressor removed via laser ablation *vs.* mock ablated males. (A) The time it takes virgin males to initiate backing along a hermaphrodite after he has been placed on a mating lawn. (B) Percentage of males successfully able to insert their spicules into a hermaphrodite. (C) Percentage of males that released sperm into the vulva. (D) Length of time from spicule insertion to retraction or popping out of the vulva. (E) Mating competition assay. *x*-axis indicates which color males had the anal depressor ablated. Yellow bars indicate females that produced only cross progeny that contained a YFP transgenic marker. Blue bars indicate females that produced only cross progeny that contained a CFP transgenic marker. Green bars indicate females that produced cross progeny that contained both YFP and CFP markers. Control n=38 females, Blue adp-ablated n=23 females, Yellow adp-ablated n=30 females. (F) Distance of spicule dorsal–ventral movement during stimulation with blue light of males grown with and without *all*-trans retinol. Each point indicates one individual male measured. Males expressed ChR2 in the anal depressor. ** *P* < 0.005, Mann–Whitney *U* test. (G) Images of spicule position in the male tail prior to and during blue light exposure. White line indicates the distance that was measured to determine total distance moved. Dorsal is to the top, posterior to the right. Scale bar = 10 µm. (H) Conceptual diagrams of the role of the anal depressor. adp, anal depressor; CFP, cyan fluorescent protein; ChR2, channelrhodopsin2; ger, gubernaculum erector; YFP, yellow fluorescent protein.

To determine more precisely what the anal depressor does during mating, we ablated this muscle in wild-type males and then mated them to 2-d-old paralyzed hermaphrodites. We found no obvious defects in mating behavior (Figure 9, A–D). This result is puzzling since the male undertakes an extensive remodeling of the muscle architecture and neural muscular connectivity, suggesting that it should play a role. Thus, we hypothesize that the adult male anal depressor is not essential for copulation but might aid in the efficiency of the behavior.

To determine if the anal depressor plays a role in mating, we set up a competition assay between intact and anal-depressor-ablated males to address who could inseminate a mate first (LeBoeuf *et al.* 2011). Intact and operated males were transgenically marked with either CFP or YFP, which allowed the determination of paternity by examination of the fluorescent progeny. One male from each group and a virgin *fog-2* female (which cannot make her own sperm) were added to a bacterial lawn. Intact and operated males were removed from the female when she was observed to have at least one egg in her uterus. To ensure that the fluorescent proteins did not indirectly disrupt mating, mock-ablated CFP- and YFP-expressing males were also tested in the competition assay; as expected, we saw no difference in the mating ability in the fluorescing males (Figure 9E). We then ablated the anal depressor in CFP males and mated them with intact YFP-expressing males, and vice versa. The competition assay indicated that intact males had a significant advantage over their anal depressor-lacking counterparts (*P* value = 0.002, Fisher’s exact test). Fifty-three percent of females were impregnated by nonablated males, 17% of females were impregnated by ablated males; in 30% we could not tell the paternity since the female gave progeny from both fathers (Figure 9E). Thus, having a functional anal depressor provides a mating advantage to males.

However, this still does not answer what is the cellular function of the male anal depressor. To address this question, we stimulated anal depressor contraction using the light-activated cation ChR2 and then observed which male structures immediately responded as a consequence; ChR2 expression was localized to the anal depressor by using the *aex-2* promoter (Mahoney *et al.* 2008). We immobilized 1-d-old virgin males using abamectin and visualized the male tails using a 100 × microscope objective lens. Upon exposure to blue light, the anal depressor did not contract in males that lacked ATR (Figure 9F). However, for males exposed to ATR, we saw their spicules shift an average of 3.3 μm in a ventral-to-dorsal movement (Figure 9, F and G). The male anal depressor left/right anterior–posterior sarcomeres are attached to the dorsal protractor muscle and the posterior proctodeal cells (Bδ.L/R, B.paa, and B.pap), the structure surrounding the sex organs (Sulston *et al.* 1980). Given this, the muscle is likely moving the spicules through its tugging on the dorsal protractor muscle (Figure 9G). The anal depressor might be used to fine-tune the spicule’s position during prodding, as the muscle displays rhythmic calcium transients during this behavior (Figure 2C). The anterior–posterior contraction of the sarcomere might also result in compressing the posterior proctodeum. This would increase the space of the proctodeal cavity, facilitating sperm passage through this structure and exit from the cloaca (Figure 9G). Taken together, this data indicates that the anal depressor precisely regulates spicule position to promote successful mating under the difficult conditions that males encounter in the wild.

## Discussion

Determining how sexually isomorphic circuits are modified to produce sexually dimorphic behaviors is a complex endeavor. Even shared systems for such basic activities like movement are modified in a sex-specific way in species ranging from *C. elegans* and *Drosophila* to humans, and this modification is dependent on the sex of the nervous system, not just differences in body morphology (Mowrey *et al.* 2014; Smith and Mittendorfer 2016; Zhang *et al.* 2016). However, little is known about the underlying mechanisms driving the sexual dimorphism.

**Figure 10:**
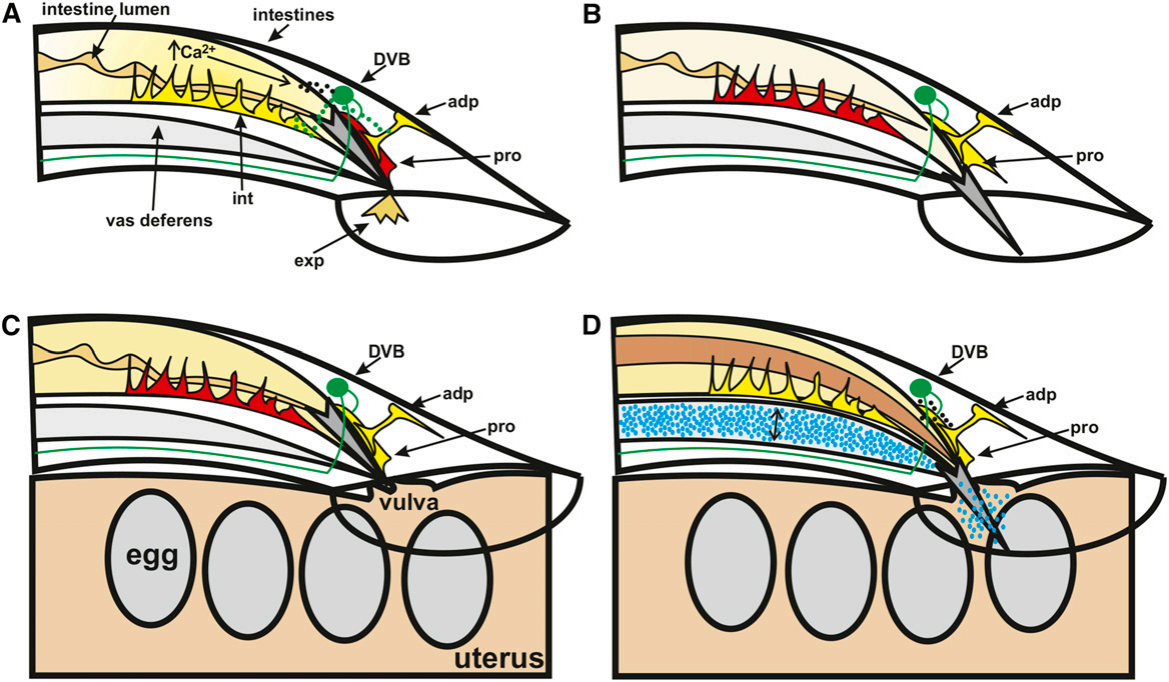
The male defecation circuit is integrated with the male mating circuit to promote copulatory behaviors. Conceptual diagram of the male tail during defecation (A and B) and mating while at the hermaphrodite vulva (C and D). Dorsal is to the top, anterior to the left. The spicule is in dark gray. The GABAergic DVB, its processes, and neurotransmitter release are in green. Active muscle are shown in yellow, nonactive muscle in red. (A) During defecation, calcium increase in the intestine results in neuropeptide (black) release that activates the DVB, which in turn activates the enteric muscle. (B) Due to electrical connections between the anal depressor and protractor, the male sex muscle are activated following the expulsion step of defecation, resulting in spicule protraction when no hermaphrodite is present. (C) The male sex muscle, including the protractor, and the anal depressor are active while the male is prodding at the hermaphrodite vulva. (D) Neuropeptide release from the DVB is necessary for spicule insertion, a process during which both the sex muscle and anal depressor contract. The intestinal muscle squeezes the intestine, possibly to support sperm (blue) movement through the vas deferens (double headed arrow); as a consequence, the intestinal lumen volume also expands (brown). adp, anal depressor; exp, waste expelled from the cloaca; GABA, γ-aminobutyric acid; int, intestinal muscle; pro, dorsal protractor.

Here, we identify ways in which the sex-shared defecation system is modified in the male to promote mating behavior. While the male defecation system still functions to expel waste, this process is arrested during mating, and the various components take on new tasks. In both sexes, the rhythmic defecation motor program is initiated by an intestinal calcium wave that activates body wall and enteric muscle and the GABAergic motor neurons AVL and DVB (Figure 10A) (Espelt *et al.* 2005; Teramoto and Iwasaki 2006; Beg *et al.* 2008; Mahoney *et al.* 2008). Due to the gap junction connections that exist between the enteric and male sex muscle (Figure 1B) (Jarrell *et al.* 2012), the sex muscle are also active during the expulsion step of defecation resulting in spicule protraction (Figure 10B). This connectivity is additionally important during mating, as the anal depressor displays similar calcium transient fluctuations to the sex muscle while the male is prodding at the hermaphrodite vulva with his spicule(s) (Figure 10C). This activity extends to the intestine and intestinal muscle upon spicule insertion, perhaps to promote sperm movement (Figure 10D). The intestinal calcium activity during mating is in contrast to its activity during defecation, where the initial Ca^2+^ transient increases occur in the intestine and trigger the defecation motor program (Espelt *et al.* 2005; Teramoto and Iwasaki 2006). We find that, during mating behavior, the movement of intestinal calcium is not as important, as males with disrupted intestinal calcium movement still display normal mating behavior. However, the role of the intestinal muscle is likely more substantial, as constipation can prevent sperm movement (Jee *et al.* 2016). Additionally, severe constipation can lead to hemorrhaging from the cloaca that results in spicule protraction (LeBoeuf and Garcia 2012). This leaves the male with no control over his spicules and therefore unable to mate. We propose that the role of the intestinal muscle during mating behavior is to prevent the intestine from inappropriately blocking sperm release (Figure 10D). The intestine might even contribute to widening the posterior to facilitate sperm movement.

Since the intestine is not coordinating defecation circuitry function during mating, this raises the question of what stimulates DVB, the anal depressor, and intestinal muscle function during this behavior. The connectivity between defecation circuitry components is altered in the male to interconnect with mating circuitry (Figure 1, A and B) (Jarrell *et al.* 2012). The hermaphrodite circuit contains gap junctions between the three enteric muscle, the intestinal, sphincter, and anal depressor, and chemical innervation by the DVB and AVL neurons (Figure 1A) (White *et al.* 1986). In the male, the anal depressor is no longer directly electrically coupled to the sphincter and intestinal muscle but instead is connected to the protractors and dorsal body wall muscle (Figure 1B) (Jarrell *et al.* 2012). In contrast, the sphincter and intestinal muscle are directly connected to the ventral body wall muscle and diagonal muscle necessary for regulating male tail positioning (Figure 1B) (Liu *et al.* 2011; Jarrell *et al.* 2012). Using calcium imaging, we showed that this connectivity is functional during mating, as the intestinal muscle, anal depressor, and male sex muscle all showed similar calcium transients that corresponded to spicule prodding and insertion. Thus, we propose that the electrical connectivity between the muscle components of the defecation and mating motor programs allow for the activity in the defecation components during mating. The DVB is also likely being activated by gap junction connectivity between the neuron and neurons regulating male mating. This neuron is electrically coupled to the HOB, involved in vulva location, and SPV, involved in sperm release (Liu and Sternberg 1995; Barr and Sternberg 1999; Jarrell *et al.* 2012; LeBoeuf *et al.* 2014). Thus, it is likely that this connectivity is activating the DVB motor neuron to promote spicule insertion.

How is neuropeptide signaling from the DVB promoting spicule insertion? The DVB no longer innervates the enteric muscle directly but instead synapses the protractor muscle and SPC and HOB neurons (Figure 1, A and B) (White *et al.* 1986; Jarrell *et al.* 2012). The SPC and protractor muscle are necessary for spicule insertion and the protractors are required for prodding as well (Liu and Sternberg 1995; Garcia *et al.* 2001). The HOB is connected to the postcloacal sensilla and is responsible for vulva location (Liu and Sternberg 1995; Barr and Sternberg 1999). While we have previously identified that cholinergic signaling plays a necessary role in promoting these behaviors (Garcia *et al.* 2001; LeBoeuf *et al.* 2007; Liu *et al.* 2011), our present results suggest that, while these behaviors still occur without neuropeptide signaling, they are not optimized, indicating that neuropeptide signaling plays a role in spicule insertion attempts, spicule penetration, and sperm transfer. This result is supported by previous work showing a role for neuropeptide signaling in turning, mate searching, and mate responsiveness aspects of the behavior (Liu *et al.* 2007; Barrios *et al.* 2008; Garrison *et al.* 2012).

In hermaphrodite defecation, the DVB makes chemical synapses to the anal depressor, activating this muscle through GABA release, resulting in expulsion (White *et al.* 1986; McIntire *et al.* 1993b). However, in the male the anal depressor is remodeled, and part of that remodeling involves the retraction of the muscle arm that connects to the DVB (Chen and García 2015). The loss of connectivity between the DVB and anal depressor is supported by the male wiring project (Jarrell *et al.* 2012). Differences in anal depressor function are also supported by ablation studies, as ablating the anal depressor in hermaphrodites results in constipation but not in males (Reiner and Thomas 1995). Here, we ablated the anal depressor in males to determine what role this muscle has on mating behavior. The anal depressor likely promotes spicule positioning and sperm release through its attachment with the posterior proctodeum, the modified rectum that contains the male sex organs.

In contrast to laser-ablating the anal depressor, loss of *unc-31*/CAPS results in severe constipation in the adult male but not the hermaphrodite. The severity of the constipation phenotype indicates that neuropeptide signaling from the DVB plays a larger role during male defecation than in hermaphrodites. Interestingly, many other components regulating the behavior in hermaphrodites do not show constipation phenotypes in the male (Reiner and Thomas 1995). This suggests that the male relies more on neuropeptide signaling during defecation, as the severe *unc-31*-induced constipation seen in the male is similar to that seen in genes that disrupt GABA synthesis and transport (Reiner and Thomas 1995). While some studies have suggested that CAPS can be involved in neurotransmitter release (Renden *et al.* 2001; Shinoda *et al.* 2016), there is no evidence that it is occurring with GABA in the DVB (Speese *et al.* 2007). GABA does play a role in regulating spicule protraction, but this is probably through male-specific neurons and muscle (Jobson *et al.* 2015). Thus, it is likely that neuropeptide signaling is promoting GABA release through a less direct mechanism.

Neuropeptide signaling has a large role in sexually dimorphic behavior across animal phyla. In *C. elegans*, several examples exist of neuropeptides released from sexually isomorphic neurons that regulate male mating. Nematocin, the *C. elegans* version of oxytocin, expressed from a single neuron in the male tail (the DVA), promotes response to potential mates and other aspects of mating behavior (Garrison *et al.* 2012). Pigment dispersing factor 1 expressed from a neuronal pair in the head promotes mate searching behavior (Barrios *et al.* 2012). Additionally, Pereira *et al.* (2015) showed that neurotransmitters can be expressed in a sexually dimorphic manner, suggesting that the same could be true of neuropeptides. In *Drosophila*, neuropeptide F, the homolog of mammalian neuropeptide Y, promotes male courtship (Lee *et al.* 2006). In mammals, neuropeptides help control important sexually dimorphic hormones that regulate both development and behavior (Ruiz-Pino *et al.* 2012). Additionally, sexually dimorphic behaviors are often controlled from brain regions that give rise to a variety of behaviors exhibited by both sexes, making identifying how shared circuitry promotes differing behavior a challenge (Bayless and Shah 2016). Using *C. elegans*, we were able to show specifically how the sex-shared neuron DVB, utilized in both sexes for defecation, additionally in the male promotes the spicule insertion step of mating behavior using neuropeptide signaling. *unc-31*/CAPS, a protein that regulates neuropeptide release, is required in the DVB to regulate spicule insertion behavior in the male. Thus, this work highlights specific circuitry modifications; animals utilize sexually isomorphic circuitry to regulate sexually dimorphic behaviors.

## Supplementary Material

Supplemental material is available online at www.g3journal.org/lookup/suppl/doi:10.1534/g3.116.036756/-/DC1.
