# Supplementary material for: Caenorhabditis elegans Male Copulation Circuitry Incorporates Sex-Shared Defecation Components To Promote Intromission and Sperm Transfer
Source: G3 (Bethesda). 2016 Dec 27;7(2):647–62. doi: 10.1534/g3.116.036756 (PMC5295609; doi:10.1534/g3.116.036756)
Supplement: Supplementary file 1 [file 647FigureS1.pdf]

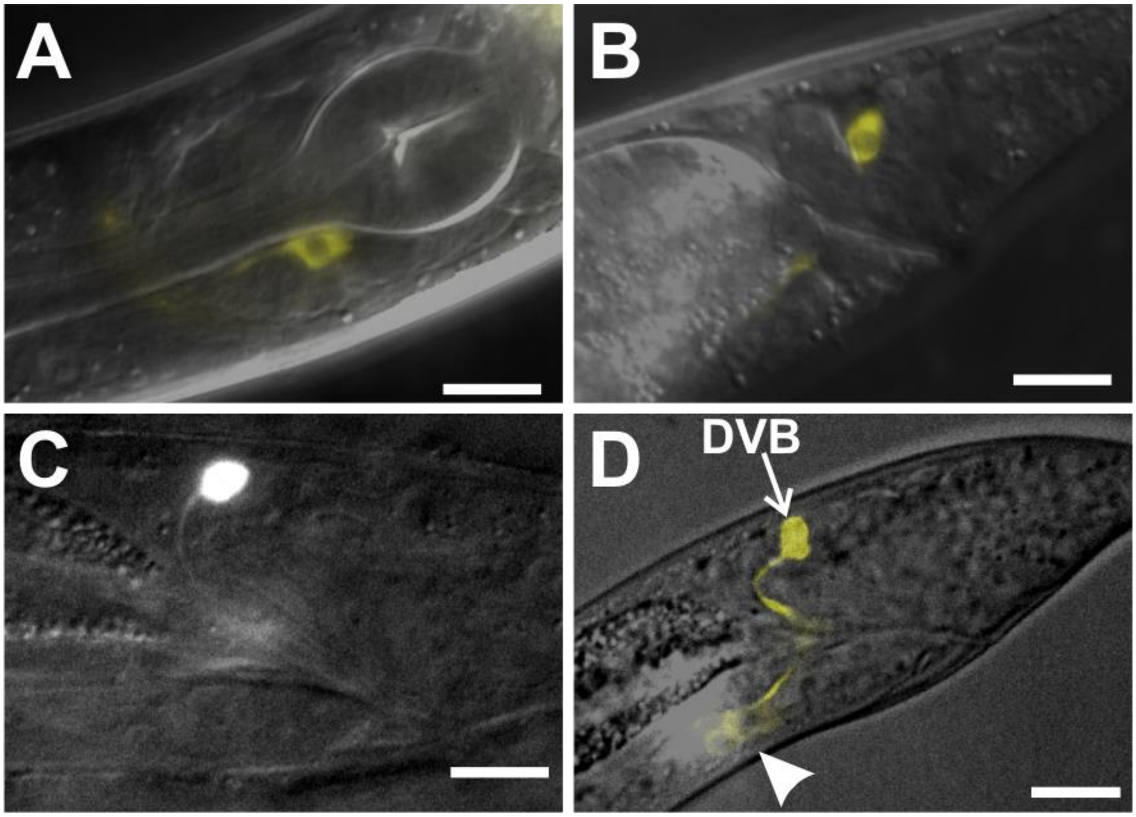

Figure S1. Robust *unc-103J* promoter expression in the DVB. Images depict Punc-103J:YFP:unc-31 expression. Dorsal is to the top, anterior to the right. (A) Variable expression in an L4 male head. The image depicts one neuron but up to three neurons can be visible on the right side near the posterior bulb. There is a process in the nerve ring that runs to the ventral cord. No processes are seen that extend to the nose. Scale bar = 10 µM. (B) Expression in an L4 hermaphrodite tail. 8/9 hermaphrodites had DVB expression; 1/9 had expression elsewhere (1 cell in the pre-anal ganglion). Scale bar = 10 µM. (C-D) Expression in an L4 male tail. 10/14 tails have expression in the DVB, 4/14 have DVB only, and 7/14 have weak, inconsistent expression in random cells in the tail. As an example, (D) shows bright expression in the DVB (arrow) and faint expression in two cells in the pre-anal ganglion (arrow head). (C) Scale bar = 10 µM. (D) Scale bar = 20 µM.
